# Is the Organ Care System (OCS) Still the First Choice With Emerging New Strategies for Donation After Circulatory Death (DCD) in Heart Transplant?

**DOI:** 10.7759/cureus.26281

**Published:** 2022-06-24

**Authors:** Mohammad Alomari, Pankaj Garg, John H Yazji, Ishaq J Wadiwala, Emad Alamouti-fard, Md Walid Akram Hussain, Mohamed S Elawady, Samuel Jacob

**Affiliations:** 1 Cardiothoracic Surgery, Mayo Clinic, Jacksonville, USA; 2 Colorectal Surgery, Mayo Clinic, Jacksonville, USA

**Keywords:** cold ischemic time, organ preservation, donation after circulatory death (dcd), donor pool expansion, organ care system, heart transplant

## Abstract

The scarcity of donor hearts continues to be a challenge in transplants for advanced heart failure patients. With an increasing number of patients on the waiting list for a heart transplant, the discrepancy in the number between donors and recipients is gradually increasing and poses a new challenge that plagues the healthcare systems when it comes to the heart. Several technologies have been developed to expand the donor pool in recent years. One such method is the organ care system (OCS). The standard method of organ preservation is the static cold storage (SCS) method which allows up to four hours of safe preservation of the heart. However, beyond four hours of cold ischemia, the incidence of primary graft dysfunction increases significantly. OCS keeps the heart perfused close to the physiological state beyond the four hours with superior results, which allows us to travel further and longer distances, leading to expansion in the donor pool. In this review, we discuss the OCS system, its advantages, and shortcomings.

## Introduction and background

End-stage organ failure remains a significant cause of mortality around the world. Among many medical breakthroughs in the 20th century, the development of solid organ transplants has been a major advancement [1]. The research and development of increasingly effective immuno-suppressive medications, tissue and organ preservation methods, major histocompatibility detection and matching, and surgical techniques have contributed to organ transplantation being a safe and reproducible operation [2].

Of all the organ failures, heart failure is responsible for the highest number of deaths worldwide [3]. End-stage heart failure is linked to a high mortality rate and an inferior quality of life. Heart transplantation remains the gold standard for the management of heart failure, with a median survival of 10 to 15 years [4]. Since the first successful human heart transplant in 1967 [5], the specialty of heart transplantation has come a long way. To begin with, in 1970, only ten centers were performing heart transplants and presently in 2021, almost 3800 heart transplants were performed in the USA alone and almost 9000 heart transplants globally (the United Network for Organ Sharing (UNOS) data) [6]. Unfortunately, due to the gradual increase in the prevalence of patients with end-stage heart failure, the donor pool cannot keep up with the demand, and many patients with advanced heart failure die while waiting for a heart transplant. Therefore, there is a constant need to increase the donor pool. Still, approximately 53% of potential donors are declined due to various reasons [7].

**Figure 1:**
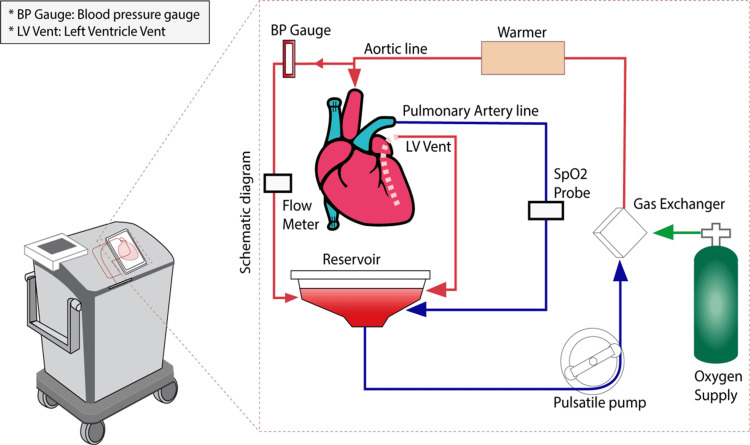
This Figure illustrates an Organ Care System (OCS) heart machine on the left with the schematic diagram on the right, breaking down the processes involved. The aorta is connected to the aortic line, the pulmonary artery is connected to the pulmonary artery line (PA line), the left atrium is opened, and the left ventricle is vented via a left ventricle vent (LV vent ). The superior and inferior vena cava are ligated. Blood from the PA line and LV vent goes to the reservoir and is pumped to the gas exchanger where it is oxygenated, passed through a warmer, and returned to the heart through the aortic line. The blood pressure, SpO2 (oxygen saturation), and flow rate are all measured during the blood circulation through the different lines. This ensures that the heart receives continuous blood circulation, and all the parameters of the heart are continuously monitored and maintained within narrow margins.

Improving organ preservation techniques can be one of the keys to increasing the donor pool [8]. One of the techniques introduced in the last two decades to improve graft useability is the organ care system (OCS). Cold static storage of the donor's heart for more than four hours leads to significant ischemic injury to the heart, which limits the duration of safe preservation, and distance of procurement; overall contributing to the poor utilization rate of accessible organ donations and has a significant impact on post-transplant survival [9]. An OCS heart is a portable extracorporeal heart perfusion and monitoring system used for the resuscitation, preservation, and assessment of donor's hearts in a near-physiologic, normothermic, and beating state intended for a potential transplant recipient. It allows continuous monitoring of aortic pressure, lactate level, and coronary blood flow of the graft [10]. Continuous measurement of lactate levels from the arterial and venous side of the graft in the OCS allows for assessing the adequacy of coronary blood flow [11] (Figure 1).

## Review

Initial work

In order to solve the drawbacks of cold static preservation, research on the ways for warm preservation was initiated in parallel. Devices used in both the approaches were similar and employed pulsatile warm perfusion of the heart with blood to allow ex-vivo recovery of the hearts with borderline function or hearts following cardiac arrest before implantation. Blood was deemed to be an ideal perfusion solution as blood has an excellent property for oxygen and nutrient delivery. In addition, blood acts as a free radical scavenger and a strong buffer against acidosis and metabolic toxicity. Also, blood protects the function of the endothelium and reduces injury [12].

It was observed for a long time that the heart recovered its function in patients dying from cardiac arrest once the patients were put on cardiopulmonary bypass prior to procurement (normothermic regional perfusion). Based on this construct, an experimental technique for harvesting donor hearts from pigs dying from cardiac arrest was developed by Fedalen and colleagues in which pigs were paralyzed using a neuromuscular blocker, disconnected from the ventilator, and were allowed to go into cardiac asystole [13]. Ensuing hypoxia caused the heart rate to drop, becoming bradycardiac and subsequently arrested. After 15 min, the heart was surgically removed and put on the ex-vivo system, which allowed blood to be perfused through the left atrium of the donor's heart. On leaving the heart, the blood enters the outflow circuit (similar to how it enters the aorta) and into a compliance chamber. The blood was oxygenated, pumped through a heater, and recirculated to the left atrium. Fresh porcine blood was used and the old blood was renewed every four hours. Researchers observed that heart function was restored to normal when the heart was connected to the OCS machine after 15 minutes of hypoxic-ischemic injury.

**Figure 2:**
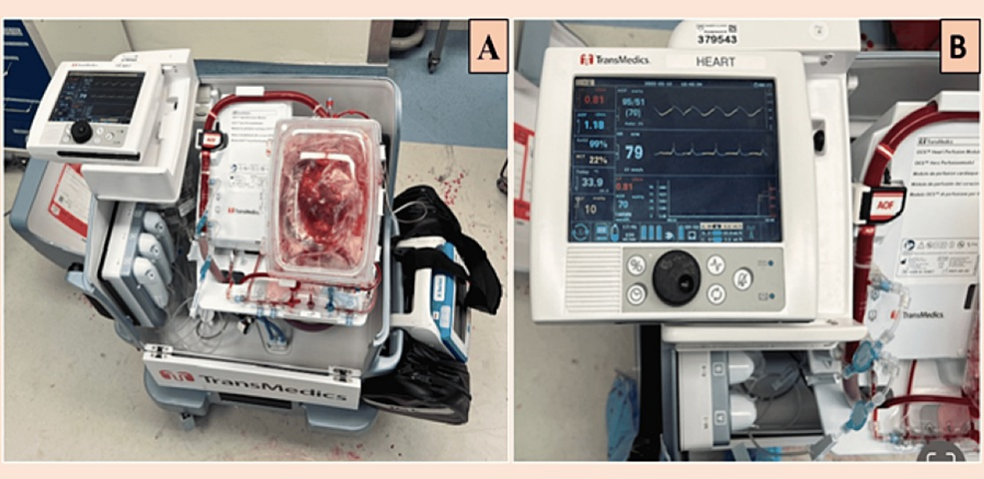
Figure shows the setup (A) and display (B) of a TransMedics Organ Care System (OCS) illustrating all the parameters of the heart that are continuously monitored. The parameters being monitored include aortic pressure (AoP), coronary flow (CF), heart rate (HR), pump flow (PF), hematocrit (HCT), mixed venous oxygen saturation percentage (SvO2), blood temperature, and lactate level. These parameters help the transplant surgeons to monitor the heart continuously and make an informed decision on the viability of the marginalized heart before transplanting it into the recipient.

A similar method was utilized during the same period but for a different purpose. Hassanein et al. [14] placed hearts in a similar device for up to 36 hours in an attempt to preserve tissue and prolong ischemic times. This attempt was made possible by recycling the system with fresh blood every six hours. Compared to hearts stored in the University of Wisconsin solution (UWS), hearts perfused continuously with the blood performed significantly better at 12 hours. Based on the concepts from these experiments, a commercially viable system was developed. It consists of a miniaturized pulsating pump with an inline heater manufactured by TransMedics (Andover, USA). The system perfuses the donor organ using blood and nutrition solution, which maintains the metabolic milieu of the heart. All parameters of the heart, including cardiac output, temperature, flow, and blood pressure, are monitored wirelessly through flat panel screens [15] (Figure 2).

How OCS works (heart from donor's chest to implantation on OCS machine)

Recovery of the Heart

The initial step in the establishment of an OCS machine for heart perfusion is the procurement of the heart. The procedure of the heart procurement varies significantly in donation after brain death (DBD) and donation after cardiac death (DCD). Cardiac death patients have a serious enough neurological injury to have a meaningful life but do not fit the criteria for brain death. In the subsequent sections, we will discuss the technique of heart procurement in the DBD and DCD donors and then attaching the heart to the OCS system. 

Donor After Brain Death

In using OCS for DBD, after sternotomy and pericardiotomy, the donor's heart is directly assessed, emphasizing the left and right ventricular contractility and the evaluation of coronary arteries by palpation and visual assessment. Then, the aortopulmonary window, superior vena cava, and inferior vena cava are dissected. When the assessment is completed, the OCS machine is prepared by unpacking the OCS disposable module and assembling the OCS machine. The procurement process begins by putting a cardioplegia cannula into the ascending aorta, and a venous drainage cannula is introduced into the right atrium. Once the patient is ready for cross-clamp, a minimum of 1,200 mL of donor blood is drawn from the right atrial cannula in a pre-heparinized bag to prime the OCS machine for the procedure. The superior vena cava is clamped, and the right atrium is vented by sharply cutting the inferior vena cava anteriorly 5 mm above the diaphragm attachment. The left or right superior pulmonary vein is severed to vent the blood returning from the lungs to prevent the left ventricular distension. Then, the ascending aorta is cross-clamped and 2,000 ml of Celsior cardioplegia is administered at a pressure of 150 mmHg in conjunction with topical hypothermia maintained with ice slush in the pericardial cradle. In our institute, we use Celsior. However, it can be different for different institutes. As we cannot monitor the coronary or aortic perfusion pressure, we deliver the cardioplegia with a pressure bag at 150 mmHg so that it is delivered at 60-80 mmHg at the aortic level. The final step in the procurement process is the donor's heart cut at the vena cavae, aorta, pulmonary arteries, and pulmonary veins.

Donor After Cardiac Death

For the donor after cardiac death (DCD), the initial step is to set up all the instruments on a separate table. Then, after injecting 30000 units of heparin, life-sustaining supports are withdrawn. As per our institutional protocol, we wait for a maximum duration of 27 minutes from the time of mean arterial pressure is below 50 mmHg for electromechanical arrest. If the donor does not have electromechanical arrest within this time, we decline the heart. If the electromechanical arrest happens within 27 minutes, and 5 minutes of wait time, the chest is entered quickly within 2-3 minutes, sternal edges are separated with a retractor, pericardium is opened. A large venous cannula inserted is into the right atrium and 1200-1500 mL of donor blood is drawn from the right atrial cannula in a pre-heparinized bag to prime the OCS machine for the procedure. Simultaneously, the OCS machine is prepared by unpacking the OCS module and assembled into the OCS machine, where it will be ready to accept the donor heart when it arrives. After collecting the donor blood, a cardioplegia cannula is inserted high into the ascending aorta. Aorta is cross clamped and 1000 ml of Del Nido cardioplegia is administered at a pressure of 150 mmHg in conjunction with topical hypothermia maintained with ice slush in the pericardial cradle. The procurement process of the donor's heart is similar to DBD procurement; however, it needs more diligence as the heart has not been mobilized earlier and is flaccid after giving cardioplegia, increasing the risk of injury to the superior and inferior vena cava, pulmonary veins, and the main and right pulmonary arteries. 

Establishment of Myocardial Perfusion on OCS Machine (Mayo Protocol)

**Figure 3:**
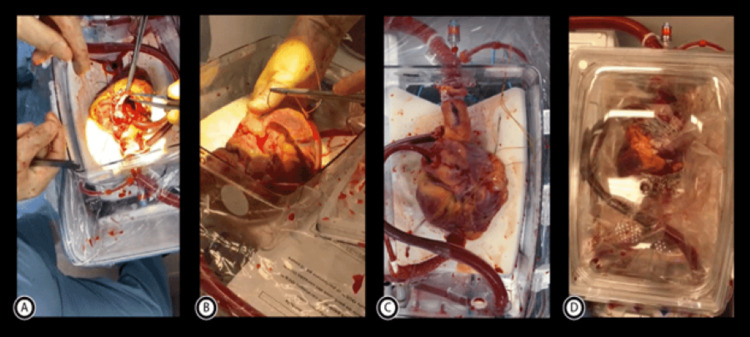
Figure illustrates the steps of the institution of myocardial perfusion on the OCS machine. The heart is placed with the left ventricle (LV) facing anteriorly and an LV vent is placed and sutured to the left atrial wall (A). after the heart starts beating, pacing wires are placed (B). After connecting the pulmonary venous cannula to its respective spout and leaving the left ventricular vent free in the tray (C), the heart is covered with sterile plastic covering and the transparent lid of the OCS (Organ Care System) tray is closed (D).

After the recovery of the heart, it is shifted to the back table. The superior vena cava is closed with a silk tie and the inferior vena cava is closed with a 4-0 polypropylene suture. Closure of the patent foramen ovale is mandatory to make the right heart a closed system for monitoring the coronary blood flow by the pulmonary artery cannula. The aortic and pulmonary cannulae are attached to the corresponding major arteries and securely fixed. The donor's heart is put on the OCS tray with the left ventricle facing anteriorly, ensuring that continuous blood flow through the aortic cannula is maintained during the procedure to de-air the aorta. The left ventricular vent is inserted through the mitral valve and fixed to the left atrial wall with a suture (Figure 3A). The pulmonary artery cannula is also vented in the OCS box cradle. Connecting the aortic cannula to the OCS machine establishes the coronary perfusion at 37℃ and after a few minutes, the heart usually starts beating. If the heart starts to fibrillate, it is shocked with the help of pads behind the heart. Ventricular wires are placed once the heart is in sinus rhythm, and ventricular pacing starts at 80 beats per minute (Figure 3B). The pulmonary arterial cannula is attached to the return spout and the inferior vena cava is repaired. As shown in Figure 3C, both aortic and pulmonary cannulae are connected to their respective spouts and the left ventricular vent is left open in the OCS tray to drain directly into the reservoir. The heart must now be maintained by having the coronary flow around 700-800 ml/min and the aortic pressure approximately 75 mmHg, which are controlled by the solution rate and aortic blood flow. The heart is covered with a sterile covering for manipulation if required during transportation and the lid of the tray is closed (Figure 3D). Regular arterial and venous blood gas measurements are taken to determine the lactate trend and potassium and calcium levels, and a periodic evaluation of the left ventricular contractility is done prior to leaving the donor hospital and during transport. Once the heart reaches the recipient OR on the OCS machine, its contractility is reassessed. Based on heart function, other hemodynamic parameters, and lactate levels, which should be less than 5 and trending down, if it is decided to proceed with the transplant, the first step is the closure of the aortic vent and disconnection of the pulmonary artery cannula from the OCS spout. It is necessary to reduce the OCS flow, clamp the aortic line and administer one liter of cold Del Nido cardioplegia via a side port. Upon completion of cardioplegia, the aortic cannula is removed from the OCS, and the heart is handed in for transplant [16].

Comparison of OCS to SCS

In the static cold storage (SCS) method, hypothermia reduces cellular metabolism and oxygen consumption to extend the organ preservation time. Once the organ is removed from the donor, the organ is placed on ice and stays in the icebox until implantation of the organ in the recipient [17-19]. However, in SCS, the donor's heart suffers from time-dependent ischemia and related reperfusion injury, which may harm cardiac function following transplantation [20]. A prolonged period of cold ischemia is a major risk factor for donor cardiac dysfunction and recipient death in transplant recipients [21]. Since 2018, the use of the SherpaPak system (Paragonix Technologies, USA) is increasing for the transport of donor hearts. SherpaPak preserves the heart in a single-use sterile disposable box, with controlled temperature ranging between 4℃ to 8℃. Utilization of this technology allows for successful transportation of hearts with predicted cold ischemic temperature and its real-time monitoring. After the publication of the first study on the safety of use of SherpaPak for the transportation of donor hearts without harmful effects [22], many clinical studies and clinical trials were initiated to evaluate the impact of using SherpaPak on postoperative outcomes. Preliminary data of the GUARDIAN (Global Utilization and Registry Database for Improved Heart Preservation) registry has shown an 8.7% increase in one-year survival rate (96.4% in the SherpaPak group vs. 88.7% in the traditional cold storage group) with the use of SherpaPak for donor heart preservation (p=0.03). Additionally, in a propensity-matched study, the incidence of post-transplant severe primary graft dysfunction (71.9% reduction (p=0.005)) and post-transplant use of acute mechanical circulatory support (AMCS) devices (38.5% reduction in all AMCS devices (p=0.03), 66.3% reduction in ECMO (extracorporeal membrane oxygenation)/ventricular assist device (VAD) use (p=0.02), and 59.7% reduction in newly placed intra-aortic balloon pump (p=0.02)) was significantly reduced with the use of the SherpaPak system [23-25]. However, whether, we can preserve the heart for longer than 4 hours without a decline in myocardial function is yet to be studied. Compared to SCS and SherpaPak both, the OCS technology allows the preservation of the heart for a longer duration and significantly reduces the total cold ischemia time. The cold ischemia time in OCS is limited to the initial and final phases, i.e., from procurement to the connection of the heart to the OCS and from disconnecting the heart from the OCS to reperfusion after the organ transplant [26].

The management of heart transplant recipients has substantially improved over the past few decades, but preservation and optimization of the graft have not received the attention they deserve. OCS can be a game-changer since the longer preservation time not only gives us the flexibility to carry out complex recipient dissections and analyze and manage the donor graft before transplant but it can also increase the pool of possible donors [27]. Listed below are the major advantages of using the OCS system for heart procurement.

Advantages

There are several advantages of using the OCS system for heart perfusion and storage during transport from the donor hospital to the recipient hospital.

1) Cold ischemia time and older age of the donors are major risk factors for morbidity and mortality post-transplant. Increased donor age is associated with a greater risk of coronary artery disease (CAD). Studies have shown that the mortality at one-year post-transplantation doubles when the cold ischemic time is increased from three hours to six hours and halved when it is less than one hour [28]. The OCS system significantly reduces the cold ischemia time of the heart and allows coronary angiography to be performed in real-time, which is the best method to diagnose CAD [29]. Also, aortic perfusion on the OCS system can act as a surrogate to diagnose significant CAD in the donor heart. In a study on the OCS system, García Sáez D et al. reported that the rising aortic pressure during heart perfusion is a predictive indicator of CAD in the donor heart. Therefore, continuous monitoring of the aortic root pressure is important because, in case an angiogram is not available, monitoring the aortic pressure and lactate aid in determining the severity of CAD and subsequently deciding on the viability of the graft [30]. A non-inferiority study about the OCS system, PROCEED II trial (prospective multicenter study) demonstrated that the outcome of heart grafts preserved with OCS heart was comparable to those preserved using the static cold storage method. The 30-day outcome in heart transplant recipients and graft survival was not affected by using the OCS for heart preservation compared to the standard cold method. Also, there was no significant difference in the incidence of high-grade rejection and length of stay in the intensive care unit between patients with a graft preserved using OCS compared to SCS [31]. Interestingly, a retrospective single-center review showed better outcomes for the patients who underwent transplants using OCS heart versus the standard cold storage method [32].

2) Post-heart transplant mortality of patients with congenital heart defects is high, especially among those who have undergone previous surgical intervention. It also remains a challenge for the surgeons to do the cardiectomy due to associated complex anatomy and possible severe adhesions from previous surgery and may need complex mediastinal dissection to explant the native heart. Further, many pediatric heart transplants may need reconstruction for complex anatomy after cardiectomy and prior to transplantation. Similarly, patients who have had prior VAD implantation may also benefit from OCS-supported donor hearts due to the anatomical complexity from a previous VAD. The preparation for transplantation can be more challenging and may take longer to do a safe cardiectomy without damaging the other structures.

The surgeon always has to wait for the donor heart to land in the vicinity of the recipient hospital before performing any irreversible step of explanting the native heart to avoid catastrophe if any unforeseen event happens. Therefore, organ transport time and time required for complex mediastinal dissection and reconstruction significantly increases the cold ischemia time of the donor's heart even after it reaches the recipient's operating room. The OCS system becomes a savior in these situations, and surgeons can perform more peaceful and conscious mediastinal dissection [27]. Fleck et al. in their study found that pediatric transplant patients had better outcomes when the donor heart was perfused with OCS than those in which SCS was used [33]. A small retrospective study by Ardehali et al reported that the use of OCS before heart transplant in patients with VAD had better 30-day survivability when compared to SCS [31]. However, due to the limited sample size, more research needs to be conducted to explore this indication in the future.

 3) The heart can be transported long distances with the OCS system, increasing the donor pool. Presently, the heart is procured within a radius of five hundred miles to keep the flight time less than two hours. However, a suitable donor or recipient may be located farther in many instances, so the organ has to be denied. With the OCS system, the heart can be procured and transported for farther distances, increasing the possibility of potentially increasing the donor pool [34].

4) OCS carries the potential for increasing the donor pool by accepting the marginal heart. Presently, hearts with left ventricular hypertrophy, EF (ejection fraction) 40-50%, downtime more than 20 minutes, and donor age >55 years are rejected due to the risk of primary graft dysfunction. However, many of these hearts have the potential to recover completely after improving the coronary perfusion and support on cardiopulmonary bypass for a short duration. The OCS system provides the opportunity to procure these marginal hearts and assess in real-time their function after establishing coronary perfusion. If the heart function improves, these hearts can undergo successful transplantation with a good outcome [35]. In the EXPAND trial, out of 93 such marginal hearts, 75 hearts were transplanted (81% utilization rate). Mean cross-clamp time and OCS perfusion time were 381 minutes and 279 minutes, respectively. In this study, the 30-day survival was 95%, the incidence of severe primary graft dysfunction within 24 hours of transplant was 11%, and the overall and graft survival at 24 months was 82% and 95%, respectively [36].

Organ care system and donor after cardiac death

Circulatory death is an irreversible cessation of all circulatory and respiratory functions [37]. While recovering the hearts from brain-dead donors (DBD), the heart is beating, and cardiac function may be examined at the time of procurement. On the other hand, hearts from donation after cardiac death (DCD) have uncertain functional status with the risk of concealed disease, and a significant risk of warm ischemic injury. Due to the difficulty in verifying the suitability of the DCD heart and the difference in the location of the donors and recipients, heart transplantation has relied primarily on DBD donations [38]. Although the first heart transplant in 1967 was a DCD donor; still, the DCD heart has remained a challenge for decades, and many institutions are working to create novel methods to safely procure DCD organs. DCD hearts already have sustained significant warm ischemia prior to perfusing with cold cardioplegia and recovery. Therefore, these hearts require a preservation strategy that reduces further ischemic insult, provides a platform for organ resuscitation and allows for graft viability assessment prior to transplantation. The OCS system allows for a better assessment of DCD heart, while SCS does not address this requirement [39]. Prior to donor heart implantation, the OCS system provides an opportunity to assess the viability of the heart by lactate level and aortic perfusion pressure [40]. Hamed et al in their prospective study evaluated OCS parameters that can best predict the post-transplant outcomes. Their study included 49 DCD hearts subjected to 30 minutes of warm ischemia followed by support with an OCS system and subsequently transplanted. Authors found that the ending lactate was the most powerful predictor of post-heart transplant graft failure. The lactate concentration in the perfusate <5 mmol/L at the end of OCS support paired with good myocardial lactate extraction and good myocardial viability [41].

Limitation

Using OCS is very expensive; each use will cost around $80,000, not including the additional cost of the hospital stay [42]. Additionally, more resources and personnel must be allocated to the application of OCS as it needs at least five staff members to work on the device each time. Another limiting factor in using OCS is the transportation of the heart from facilities. OCS transportation is more complicated than SCS because there is a need for upgraded cars and airplanes as the OCS machine must be plugged into an outlet and connected to Wi-Fi [43]. Recently, normothermic regional perfusion has arrived as a competitor to OCS. Although it is much more cost-effective, it still needs a large number of staff members and needs OCS for long-distance preservation of cardiac function.

## Conclusions

The OCS holds the potential for increasing the donor pool by increasing the number of DCD hearts, procurement of marginal hearts, and long-distance procurement, which will help distant transplant centers to receive donor hearts. The OCS will help the organ donor pool to expand and increase the number of hearts available for transplantation. However, it needs to be more cost-effective and available for more centers around the country.
